# SLC25A11 Is Associated with KDM2A-Dependent Reduction in rRNA Transcription Induced by Aminooxyacetic Acid

**DOI:** 10.3390/cells14211655

**Published:** 2025-10-22

**Authors:** Yuji Tanaka, Nagisa Miyazawa, Yuuki Toba

**Affiliations:** Laboratory of Transcriptional Regulation, Faculty of Pharmacy, Takasaki University of Health and Welfare, 60 Nakaorui-machi, Takasaki-shi 370-0033, Gunma, Japan

**Keywords:** K-demethylase 2A (KDM2A), malate–aspartate shuttle (MAS), ribosomal RNA (rRNA), SLC25A11

## Abstract

**Highlights:**

**What are the main findings?**

- Malate–aspartate shuttle (MAS) inhibitor AOA induces KDM2A-dependent reduction of rRNA transcription, whereas another MAS inhibitor, NPM, does not.
- SLC25A11, the αKG/malate carrier in MAS, is associated with KDM2A activity under MAS inhibition.

**What are the implications of the main findings?**

- The induction capacity of MAS inhibitor to induce KDM2A activity differs depending on the inhibition point.
- SLC25A11 function has potential for modulation of epigenetic regulation.

**Abstract:**

The malate–aspartate shuttle (MAS) is an NADH shuttle that transports cytoplasmic reducing equivalents to the mitochondria for producing energy. We previously demonstrated that K-demethylase 2A (KDM2A), a jmjC-type histone demethylase, decreases ribosomal RNA (rRNA) transcription via demethylation of H3K36me2 in the rRNA gene promoter region in response to energy reduction in MCF-7 cells. However, whether MAS inhibition is involved in KDM2A activity has not been investigated. In this study, we demonstrate that aminooxyacetic acid (AOA), which inhibits aspartate transaminase (AST/GOT) in MAS, decreased intracellular ATP levels and reduced rRNA transcription via KDM2A-dependent reduction in H3K36me2 levels in the rRNA gene promoter in MCF-7 cells. On the other hand, N-phenylmaleimide (NPM), which inhibits the mitochondrial αKG/malate carrier SLC25A11 in MAS, also decreased intracellular ATP levels but did not induce KDM2A activity. Additionally, NPM pretreatment or knockdown of SLC25A11 inhibited AOA-induced KDM2A activity. Dimethyl αKG, a cell-permeable αKG, restored KDM2A activity inhibited by NPM-pretreatment in AOA-treated cells. These results demonstrate that AOA and NPM have different abilities to induce a decrease in rRNA transcription via KDM2A. Furthermore, the αKG/malate carrier SLC25A11 is associated with KDM2A-dependent reduction in rRNA transcription via demethylation under MAS inhibition.

## 1. Introduction

The NADH shuttle plays an important role in energy production during aerobic respiration [1]. The reducing equivalents of NADH produced in cytoplasm are transferred to the mitochondria via the NADH shuttle and used for ATP synthesis through the electron transport chain (ETC). The malate–aspartate shuttle (MAS) is an NADH shuttle and consists of four reactions and two carriers, controlling the indirect transport of reducing equivalents and the recycling of intermediates and regenerating NAD^+^ used in glycolysis [2,3]. Among these, aspartate aminotransferase (AST/GOT) is involved in the reaction that transfers the amino group of aspartate to α-ketoglutarate (αKG) in the cytoplasm, and in the reaction that transfers the amino group of glutamate to oxaloacetate in the mitochondria. On the other hand, 2-oxoglutarate (αKG)/malate carrier (OGC/SLC25A11) is involved in the exchange transport of malate from the cytoplasm to the mitochondria and of αKG from the mitochondria to the cytoplasm [2]. MAS links other metabolic pathways such as glycolysis and ETC. The defection of MAS enzymes and carriers alters metabolism, including glycolysis, and causes several diseases with metabolic or energy abnormalities [2,3,4]. This is likely related to metabolic abnormalities and downstream responses caused by MAS dysfunction. However, the downstream responses by MAS dysfunction have not been sufficiently elucidated. Especially, while it has been reported that MAS activity contributes to histone acetylation [5], how MAS dysfunction is involved in epigenetic regulation remains unclear.

Histone-modifying enzymes use metabolic intermediates as cofactors, and their levels affect enzyme activities [6,7,8]; jmjC-type histone demethylases require a side reaction from αKG to succinate and affect various biological events through chromatin regulation [9,10,11,12]. Previously, we showed that K-demethylase 2A (KDM2A), a jmjC-type demethylase, reduced rRNA transcription via demethylation of di-methylated histone H3 lysine 36 (H3K36me2) in the ribosomal RNA (rRNA) gene promoter region in the breast cancer cell line MCF-7 in response to starvation [13]. This KDM2A-mediated reduction was also induced by 2-deoxyglucose (2-DG), metformin, or gallic acid [14,15,16]. In these studies, KDM2A-mediated reduction was induced within 4 h after treatment, and the activation of AMP-activated protein kinase (AMPK), a cellular energy sensor, was necessary for this induction. The process of constructing ribosomes and subsequent translation consumes large amounts of cellular energy and resources [17,18]. The transcription of rRNA is the first step in ribosome assembly and associated with protein synthesis and subsequent cell proliferation [19,20]. Indeed, 2-DG treatment reduced cell proliferation via KDM2A in MCF-7 cells [14]. These observations suggest that the control of rRNA transcription by KDM2A is involved in the response to environmental changes such as energy reduction. However, whether MAS inhibition affects KDM2A activity has not been investigated.

This study was aimed at examining whether MAS inhibition induces epigenetic regulation mediated by KDM2A using aminooxyacetic acid (AOA), an AST/GOT inhibitor [21,22], and N-phenylmaleimide (NPM), an OGC/SLC25A11 inhibitor [23,24]. We analyzed the role of KDM2A in MAS inhibition using MCF-7 cells, in which KDM2A-dependent regulation is induced in response to energy reduction. We show that both AOA and NPM treatments reduced ATP levels in MCF-7 cells. However, KDM2A-mediated reduction in rRNA transcription through decreased H3K36me2 levels in the rRNA gene promoter region was induced by AOA but not by NPM. These results indicate that MAS inhibitor induces epigenetic regulation of rRNA genes via KDM2A, but their induction capacity varies depending on the inhibition target. Additionally, NPM pretreatment or SLC25A11 knockdown inhibited AOA-induced KDM2A activity. Dimethyl αKG (DMαKG), a cell-permeable αKG, restored KDM2A activity inhibited by NPM-pretreatment in AOA-treated cells. These results suggest that the αKG/malate carrier SLC25A11 is associated with KDM2A-dependent demethylation under MAS inhibition by AOA. Collectively, these findings indicate that the αKG/malate transporter is associated with epigenetic control by KDM2A during MAS inhibition and may alter downstream regulation.

## 2. Materials and Methods

### 2.1. Chemicals

AOA (Sigma-Aldrich Co., LLC., Burlington, MA, USA; #C13408) was dissolved in water at a concentration of 1 M to prepare a stock solution. NPM (Tokyo Chemical Industry Co., Ltd., Tokyo, Japan; #P0900) was dissolved in ethanol to 0.1 M as a stock solution. Dimethyl αKG (DMαKG) (TCI; #K0013) was also dissolved in DMSO to 1 M as a stock solution.

### 2.2. Antibodies

Anti-phosphorylated AMPKα [Thr-172; Cell Signaling Technology (CST), Inc., Danvers, MA, USA; #2535], anti-AMPKα (CST; #5831), and anti-SLC25A11 (Proteintech Group, Inc., Rosemont, IL, USA; #12253-1-AP) were used for immunoblotting. Anti-KDM2A (Proteintech; #24311-1-AP) was used for immunoblotting and chromatin immunoprecipitation (ChIP) assays. Anti-dimethyl histone H3 [Lys36; MAB Institute Inc. (MAB), Kanagawa, Japan; #MABI0332-100], anti-trimethyl histone H3 (Lys36; MAB; #MABI0333-100), anti-histone H3 (MAB; #MABI0001-100), and a control normal mouse IgG (Santa Cruz Biotechnology, Inc., Dallas, TX, USA; #sc-3878) were used for the ChIP assay.

### 2.3. Cell Culture

MCF-7, a human breast cancer cell line, was cultured in RPMI 1640 medium (Nacalai Tesque Inc., Kyoto, Japan; #30264-85) with added 10% fetal calf serum and 1% penicillin-streptomycin mixed solution (Nacalai Tesque; #09367-34) and maintained at 37 °C and 5% CO_2_ under humidified conditions.

### 2.4. Gene Knockdown

Cells were transfected with siRNA using Lipofectamine™ RNAi MAX reagent (Thermo Fisher Scientific, Waltham, MA, USA; #13778150) according to the manufacturer’s instructions. The siRNA sequence for KDM2A knockdown was 5′-GAACCCGAAGAAGAAAGGAUUCGUUU-3,’ which was used and validated in a previous study [19,21]. The sequences of siRNAs, which were purchased from Thermo Fisher Scientific, were 5′-CCUUGGCAUCUACCGUGCUGUUUUU-3′ for SLC25A11#1 and 5′-CCUGAAUCCGAACAUGUGUGGAUGAUUU-3′ for SLC25A11#2. The Stealth™ RNAi Negative Control Medium GC Duplex (Thermo Fisher Scientific; #12935300) was used as a control siRNA.

### 2.5. Measurement of Intracellular ATP Levels

The ATP levels were analyzed with a luciferase assay system using a Cell Titer-Glo 2.0 assay^®^ kit (Promega Corporation, Madison, WI, USA; #G9241). Briefly, 2000 cells per well were plated in a 96-well white plate. The following day, drug treatment was performed as indicated in each figure, and the cells were used for the assay according to the manufacturer’s instructions.

### 2.6. Total RNA Isolation and Reverse Transcription-Quantitative PCR

Total RNA from cells treated under each condition was isolated using a NucleoSpin RNA Plus kit (Takara Bio Inc., Shiga, Japan; #740984), according to the manufacturer’s instructions; cDNA was synthesized from the same quantity of total RNA using FastGene^®^ Scriptase II cDNA Synthesis 5x ReadyMix (Nippon Genetics Co., Ltd., Tokyo, Japan; #NE-LS64). After cDNAs had been diluted with water to a suitable concentration, the samples were analyzed by quantitative real-time PCR (qRT-PCR) using KOD SYBR qPCR Mix reagent (Toyobo Inc., Osaka, Japan; #QKD-201) or a KAPA SYBR Fast qPCR kit (Nippon Genetics; #KK4602) and the CFX Connect real-time PCR detection system (Bio-Rad Laboratories, Inc., Hercules, CA, USA) or LightCycle 96 system (Nippon Genetics; #05815916001). The expression levels were normalized to the β-actin mRNA level in each sample and are shown as fold changes relative to the control condition. Pre-rRNA levels, the first product of rRNA transcription, were measured to evaluate rRNA transcription levels. The primers for human pre-rRNA were 5′-GCTGACACGCTGTCCTCTG-3′ and 5′-TCGGACGCGCGAGAGAAC-3′. Primers for human KDM2A mRNA were 5′-TCCCCACACACATTTTGACATC-3′ and 5′-GGGGTGGCTTGAGAGATCCT-3′. Primers for human β-actin mRNA were 5′-CGTCTTCCCCTCCATCGT-3′ and 5′-GAAGGTGTGGTGCCAGATTT-3′. Primers for human SLC25A11 mRNA were 5′-TCAGCGGTCTTGTCACCAC-3′ and 5′-CAGCCCGTTCTTGTATTCCG-3′. Primer sequences for SLC25A11 mRNA were received from PrimerBank [25].

### 2.7. Chromatin Immunoprecipitation Assay

ChIP assays based on Dynabeads^®^ protein G (Thermo Fisher Scientific; #10003D) and Chelex 100 (Bio-Rad Laboratories, Inc.; #1421253) DNA purification were performed using the same methods as those in previous studies [16]. DNA fragments collected by the ChIP assay were quantified using qRT-PCR. The primers used to detect the rRNA gene promoter region (+1 to +155 bp from the transcription start site) were the same as those used to detect pre-rRNA, as described above. To assess the specific binding level, the quantity predicted by qRT-PCR in the collected samples was divided by that from the input amplification, and the quantity predicted by the control antibody (normal mouse IgG) analysis was subtracted. For histone modification analysis, the value subtracted by the control IgG antibody was further normalized to the value of the histone H3 antibody. The fold changes and standard deviations obtained via these analyses are shown in the figures.

### 2.8. Immunoblotting

After each treatment, the cells were washed with phosphate-buffered saline and collected by scraping. Cells were lysed in dye- and reducing agent-free SDS-PAGE sampling buffer (100 mM Tris, pH 6.8, 4% SDS and 20% glycerol). The concentration of the lysate was measured using a Protein Assay Bicinchoninate Kit (Nacalai Tesque; #06385) with a ten-fold diluted lysate. After adding the dye and dithiothreitol to the lysate, the extracts were adjusted to the same concentrations as that of the sampling buffer. The lysates were subjected to SDS-PAGE using an Extra PAGE One Precast Gel (Nacalai Tesque; # 13062-84) or self-made gel and transferred onto a polyvinylidene fluoride membrane (Millipore, Burlington, MA, USA; #IPVH00010). After blocking with skim milk, the membrane was incubated with specific antibodies. Protein bands were detected using Chemi-Lumi One Super (Nacalai Tesque; # 02230) and the Amersham™ Imager 600 system (Cytiva, Tokyo, Japan). The band intensity was measured using the ImageJ software version 1.54p.

### 2.9. Cell Proliferation

To analyze cell numbers following drug treatment, cells were collected and counted using a Burker–Turk-type hemocytometer. The averages obtained from triplicate measurements were calculated, and the fold changes and standard deviations are shown in the figures.

### 2.10. Statistical Analysis

The number of trials from independent experiments is shown in the figure legends. Error bars indicate the standard deviations in each figure. *p*-values were calculated using one-way analysis of variance with Tukey’s multiple comparison test between each group using the EZR software version 1.62 [26].

## 3. Results

### 3.1. AOA or NPM Treatments Decrease ATP Levels and Activate AMPK

**Figure 1:**
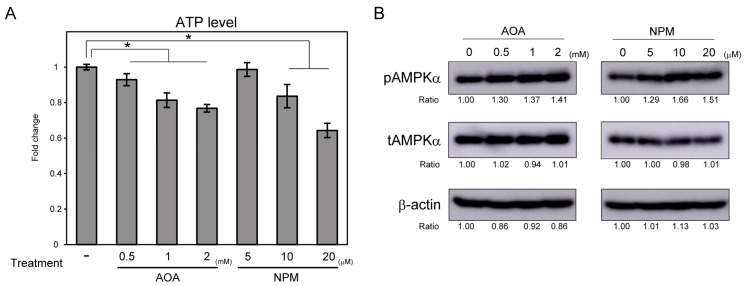
AOA and NPM treatments decrease ATP levels and activate AMPK. (**A**) AOA and NPM decrease ATP levels. ATP levels of MCF-7 cells treated with AOA and NPM at the indicated concentrations for 4 h were measured. Relative ATP levels are shown as fold changes relative to non-treated cells. Standard deviations are shown. *n* = 4, *; *p* < 0.05. (**B**) AOA and NPM activate AMPK. MCF-7 cells treated with AOA and NPM at the indicated concentrations for 4 h were analyzed by immunoblotting using antibodies for phosphorylated AMPKα (pAMPKα), total AMPK (tAMPKα), and β-actin. Ratios of the band densities relative to the non-treatment are shown.

AOA and NPM, which inhibit AST/GOT and OGC/SLC25A11, respectively, have been reported to reduce intracellular ATP levels [23,27]. Therefore, we first confirmed whether short-term treatment with AOA or NPM reduce intracellular ATP levels in MCF-7 cells. Decreases in ATP levels were detected 4 h after treatment with 1 mM AOA or 10 μM NPM (Figure S1). When MCF-7 cells were treated with 0.5, 1, or 2 mM AOA and 5, 10, or 20 μM NPM for 4 h, 5 μM NPM hardly decreased ATP levels. However, increasing concentrations of AOA and NPM resulted in a dose-dependent decrease in ATP levels (Figure 1A). After 4 h of treatment, a similar reduction in ATP levels was detected upon treatment with 10 μM NPM and 1 mM AOA (Figure 1A). AOA and NPM also increased the levels of phosphorylated AMPKα (Thr172), a marker of AMPK activation (Figure 1B). In contrast, total AMPKα and β-actin levels were comparable (Figure 1B). These results suggest that AOA and NPM reduce intracellular ATP levels and activate AMPK.

### 3.2. AOA Treatment Reduced H3K36me2 Levels in the rRNA Gene Promoter Region and rRNA Transcription via KDM2A

**Figure 2:**
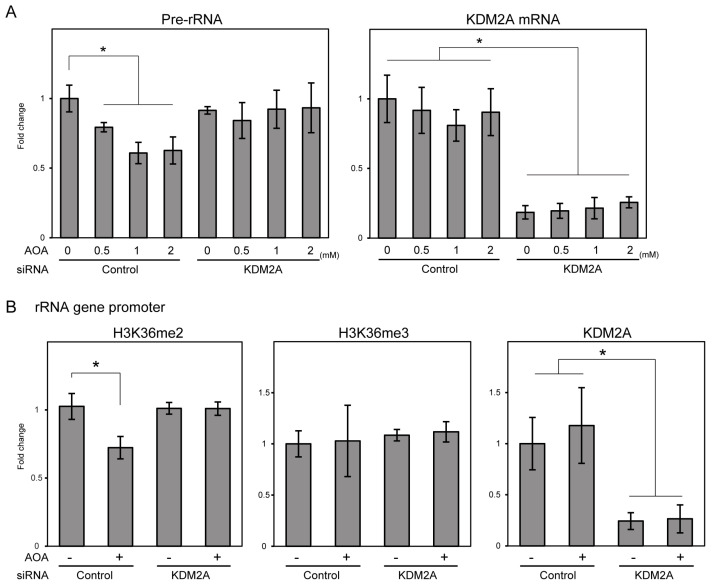
AOA decreased pre-rRNA and H3K36me2 levels in the rRNA gene promoter region through KDM2A. (**A**) AOA treatment decreases pre-rRNA levels through KDM2A. Pre-rRNA and KDM2A mRNA levels in MCF-7 cells transfected with control siRNA (Control) or siRNA for KDM2A (KDM2A) treated with AOA at the indicated concentrations for 4 h were analyzed using RT-qPCR. Levels of pre-rRNA and KDM2A mRNA were normalized to β-actin mRNA levels and are shown as fold changes relative to control cells without AOA treatment. (**B**) AOA treatment reduces H3K36me2 levels in the rRNA gene promoter region through KDM2A. H3K36me2, H3K36me3, and KDM2A levels in the rRNA gene promoter region of MCF-7 cells transfected with control siRNA (Control) or siRNA for KDM2A (KDM2A), treated with or without 1 mM AOA for 4 h, were analyzed using the ChIP assay. Levels of H3K36me2 and H3K36me3 were normalized to total histone H3 levels and are shown as fold changes relative to control cells without AOA treatment. Standard deviations are shown. *n* = 3, *; *p* < 0.05.

**Figure 3:**
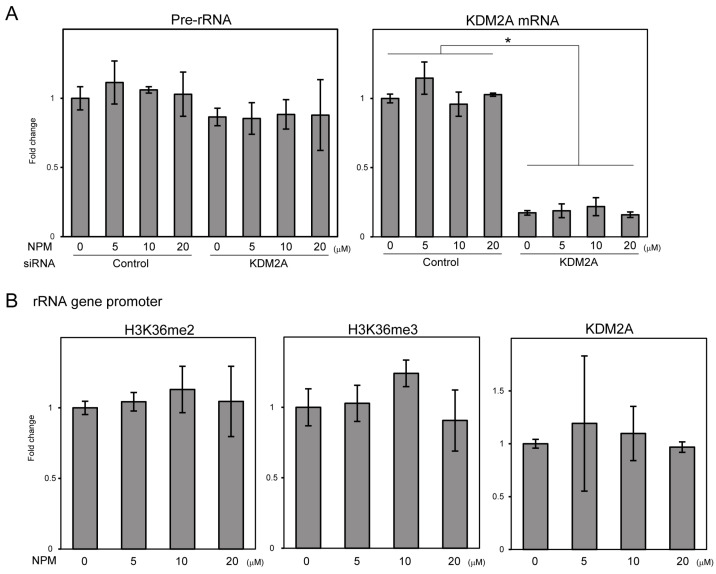
NPM did not induce KDM2A-mediated reductions in pre-rRNA and H3K36me2 levels in the rRNA gene promoter region. (**A**) NPM treatment did not decrease pre-rRNA levels. Pre-rRNA and KDM2A mRNA levels of MCF-7 cells transfected with control siRNA (Control) or siRNA for KDM2A (KDM2A) treated with NPM for 4 h were analyzed using RT-qPCR. Fold changes relative to non-treated cells are shown. (**B**) NPM treatment did not reduce H3K36me2 levels in the rRNA gene promoter region. H3K36me2, H3K36me3, and KDM2A levels in the rRNA gene promoter region of MCF-7 cells treated with NPM for 4 h were analyzed using the ChIP assay. Fold changes relative to non-treated cells are shown. Standard deviations are shown. *n* = 3, *; *p* < 0.05.

We have previously shown that AMPK activation by 2-DG is involved in KDM2A activity, which decreases rRNA transcription through demethylation of H3K36me2 in the rRNA gene promoter region [14]. Therefore, we tested whether AOA (Figure 2) and NPM (Figure 3) induce KDM2A activity. Treatment of MCF-7 cells transfected with control siRNA with 0, 0.5, 1, and 2 mM AOA for 4 h reduced pre-rRNA levels, the first product of rRNA transcription, in a dose-dependent manner (Figure 2A). The reduction in pre-rRNA levels by 1 mM AOA was not detected in cells transfected with siRNA for KDM2A, which has been validated in previous studies (Figure 2A) [13,14]. KDM2A levels were not significantly altered by AOA in control cells. KDM2A knockdown by siRNA was also confirmed (Figure 2A). Levels of H3K36me2, a specific substrate of KDM2A, were reduced by 1 mM AOA treatment for 4 h in the rRNA gene promoter region in control cells, but not in KDM2A knockdown cells (Figure 2B). AOA treatment alone did not significantly alter KDM2A levels in the rRNA gene promoter region (Figure 2B). We confirmed that KDM2A knockdown reduced KDM2A levels in that region (Figure 2B). Levels of H3K36me3, which is not a direct substrate of KDM2A, were not altered by these treatments (Figure 2B). These results suggest that AOA induced the inhibition of rRNA transcription via demethylation of H3K36me2 in the rRNA gene promoter region dependent on KDM2A. On the other hand, in the rRNA gene body region, the levels of H3K36me2, H3K36me3, and KDM2A were not altered by AOA (Figure S2). This suggests that demethylation by KDM2A occurs in a promoter-specific manner. Additionally, AMPK inhibitor prevented AOA-induced repression of rRNA transcription and H3K36me2 reduction in the rRNA gene promoter region (Figure S3). These results are similar to those observed in cells treated with 2-DG in a previous study [14] and suggest that AOA induces a response similar to energy reduction to control KDM2A activity. Furthermore, GOT1 knockdown also induced a decrease in the levels of pre-rRNA and H3K36me2 in the rRNA gene promoter region dependent on KDM2A, similar to AOA treatment (Figure S4). These results suggest that induction of KDM2A activity by AOA treatment was caused by GOT1 inhibition.

### 3.3. NPM Treatment Does Not Reduce rRNA Transcription and H3K36me2 Levels in the rRNA Gene Promoter Region

Next, we analyzed NPM treatment. NPM treatment with 10 and 20 μM decreased ATP levels and activated AMPK (Figure 1B). However, pre-rRNA levels did not decrease in MCF-7 cells treated with 10 and 20 μM NPM for 4 h (Figure 3A). Knockdown of KDM2A reduced the levels of KDM2A mRNA but did not affect pre-rRNA levels in NPM-treated cells (Figure 3A). The levels of H3K36me2, H3K36me3, and KDM2A in the rRNA gene promoter region were not decreased by NPM treatment (Figure 3B). These levels were also not significantly altered in the rRNA gene body region upon NPM treatment (Figure S5). These results suggest that, unlike AOA treatment, NPM treatment cannot induce KDM2A activity to reduce rRNA transcription via demethylation. 

### 3.4. AOA and NPM Reduce MCF-7 Cell Proliferation

**Figure 4:**
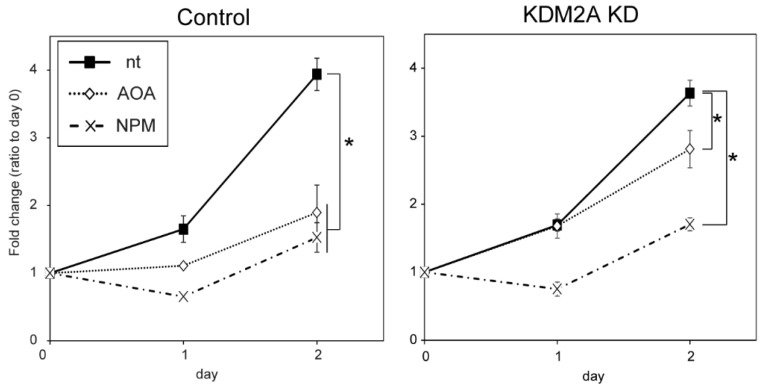
AOA treatment decreased cell proliferation via KDM2A, while NPM decreased cell proliferation independently of KDM2A. The number of MCF-7 cells transfected with control siRNA or siRNA for KDM2A treated with 1 mM AOA, 10 μM NPM, or without either reagent for 24 and 48 h was quantified. Averages of fold changes relative to day 0 are plotted with standard deviations. *n* = 3, *; *p* < 0.05.

Previously, 2-DG treatment inhibited proliferation of MCF-7 cells in a KDM2A-dependent manner [14]. To further analyze the differences between AOA and NPM, we analyzed the proliferation of MCF-7 cells treated with 1 mM AOA and 10 μM NPM, which induced a similar reduction in ATP levels (Figure 1A). Both treatments for 2 days reduced the numbers of MCF-7 cells transfected with control siRNA (Figure 4). KDM2A knockdown weakened the AOA-mediated decrease, whereas it did not alter the NPM-mediated decrease (Figure 4). These results suggest that AOA induces KDM2A-dependent inhibition of cell proliferation, while NPM induces KDM2A-independent inhibition.

### 3.5. NPM Pretreatment Abolishes AOA-Mediated Reductions in rRNA Transcription and H3K36me2 Levels in the rRNA Gene Promoter Region

**Figure 5:**
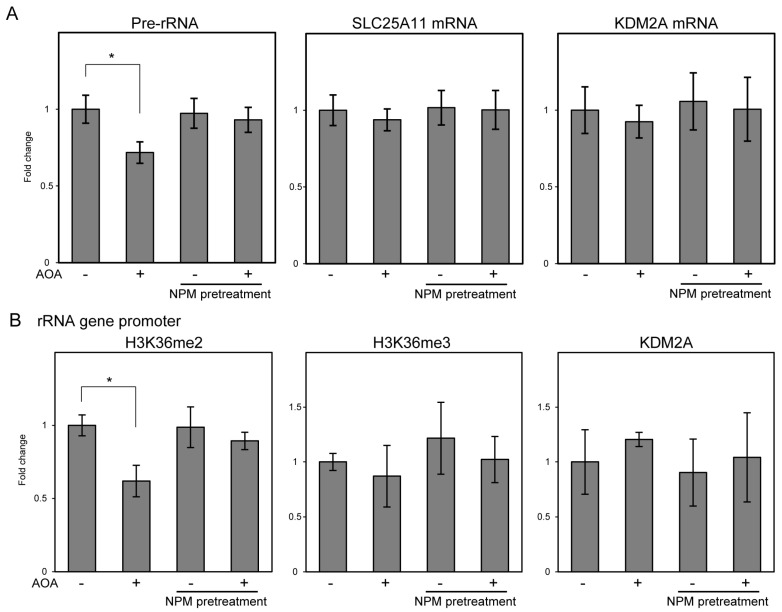
NPM pretreatment inhibits the AOA-mediated reduction in pre-rRNA and H3K36me2 levels in the rRNA gene promoter region. (**A**) NPM pretreatment inhibited AOA-mediated pre-rRNA reduction. MCF-7 cells were treated with 1 mM AOA for 4 h. Additionally, 10 μM NPM pretreatment was performed 1 h before AOA treatment. The levels of pre-rRNA, SLC25A11 mRNA, and KDM2A mRNA were analyzed using RT-qPCR. Fold changes relative to non-treated cells are shown. (**B**) NPM pretreatment inhibited AOA-mediated reduction in H3K36me2 levels in the rRNA gene promoter region. MCF-7 cells were treated under the same condition as that used in (**A**). H3K36me2, H3K36me2, and KDM2A levels in the rRNA gene promoter region were analyzed using the ChIP assay. Fold changes relative to non-treated cells are shown. Standard deviations are shown. *n* = 3, *; *p* < 0.05.

NPM treatment induced energy reduction similar to AOA treatment but failed to induce KDM2A activity to reduce rRNA transcription (Figure 1, Figure 2 and Figure 3). This suggests that NPM may exert functions that inhibit KDM2A activity. To test this, we investigated whether NPM suppresses KDM2A activity induced by AOA. Similarly to the above results, 1 mM AOA decreased pre-rRNA levels, but pretreatment with 10 μM NPM 1 h before AOA treatment abolished this reduction (Figure 5A). The mRNA levels of SLC25A11, the target of NPM, and KDM2A were not significantly altered by these treatments (Figure 5A). H3K36me2 levels in the rRNA gene promoter region were also decreased by AOA and abolished by NPM pretreatment (Figure 5B). H3K36me3 and KDM2A levels were not significantly altered (Figure 5B). These results suggest that NPM pretreatment abolished AOA-induced reduction in rRNA transcription, which was mediated by KDM2A.

### 3.6. SLC25A11 Is Involved in AOA-Mediated Reductions in rRNA Transcription and H3K36me2 Levels in the rRNA Gene Promoter Region

**Figure 6:**
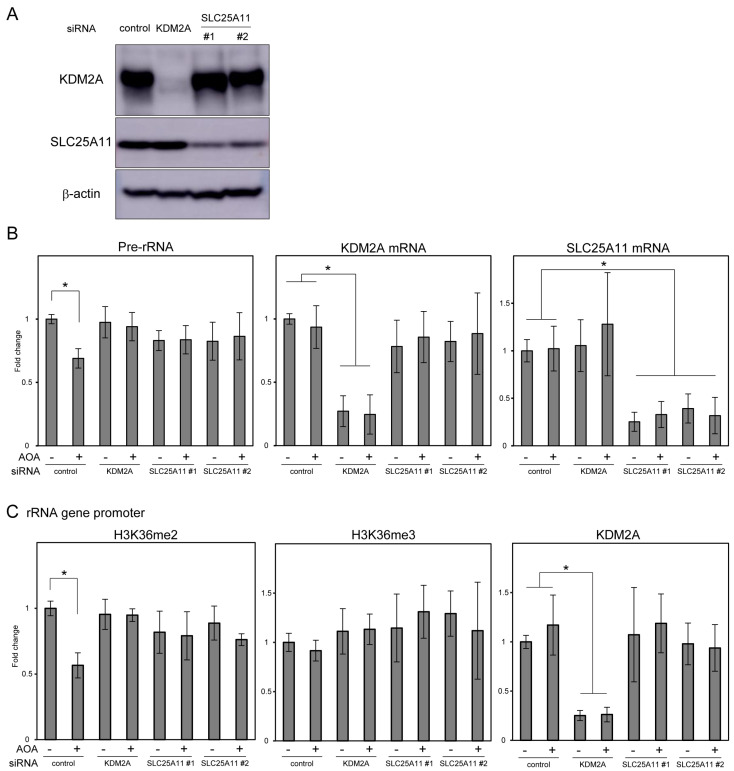
SLC25A11 knockdown inhibits the AOA-mediated reduction in pre-rRNA and H3K36me2 levels in the rRNA gene promoter region. (**A**) Knockdown of KDM2A and SLC25A11. MCF-7 cells were transfected with control siRNA (Control), siRNA for KDM2A (KDM2A), or siRNA for SLC25A11 #1 or #2 and analyzed using immunoblotting with specific antibodies for KDM2A, SLC25A11, and β-actin. (**B**) SLC25A11 knockdown abolishes the AOA-mediated decrease in pre-rRNA levels. MCF-7 cells transfected with control siRNA, siRNA for KDM2A, or siRNAs for SLC25A11 #1 and #2 were treated with 1 mM AOA for 4 h. The levels of pre-rRNA, KDM2A mRNA, and SLC25A11 mRNA were analyzed using RT-qPCR. Fold changes relative to control cells without AOA treatment are shown. (**C**) SLC25A11 knockdown abolishes the AOA-mediated decrease in H3K36me2 levels in the rRNA gene promoter region. MCF-7 cells transfected with indicated siRNAs and treated under the same condition as that used in (**B**). H3K36me2, H3K36me3, and KDM2A levels in the rRNA gene promoter region were analyzed using the ChIP assay. Fold changes relative to control cells without AOA treatment are shown. Standard deviations are shown. *n* = 5 (**B**). *n* = 3 (**C**), *; *p* < 0.05.

To clarify whether SLC25A11 is associated with the reduction in rRNA transcription induced by AOA, we analyzed SLC25A11-knockdown cells. SLC25A11 protein levels were reduced by siRNA for SLC25A11 #1 and #2, while KDM2A levels were hardly altered by these siRNAs (Figure 6A). We also confirmed that siRNA for KDM2A decreased KDM2A levels, while it hardly altered SLC25A11 levels (Figure 6A). Consistent with these results, treatment with 1 mM AOA for 4 h reduced pre-rRNA levels in control cells, which was abolished by KDM2A knockdown (Figure 6B). Treatment with siRNA for SLC25A11 #1 or #2 also abolished AOA-induced pre-rRNA reduction (Figure 6B). These knockdown effects were also confirmed by the mRNA levels of KDM2A and SLC25A11 (Figure 6B). H3K36me2 levels in the rRNA gene promoter region were also decreased by AOA treatment in control cells (Figure 6C). In contrast, SLC25A11 knockdown abolished AOA-mediated decreases in H3K36me2 levels, as did KDM2A knockdown (Figure 6C). H3K36me3 and KDM2A levels in the rRNA gene promoter region were not significantly altered by these treatments (Figure 6C). Considering that AOA-mediated reduction in rRNA transcription occurred in a KDM2A-dependent manner (Figure 2), these results suggest that SLC25A11 contributes to KDM2A activity to reduce rRNA transcription under AOA treatment.

### 3.7. DMαKG Restored the Reduction in rRNA Transcription Inhibited by NPM Pretreatment in AOA-Treated Cells

**Figure 7:**
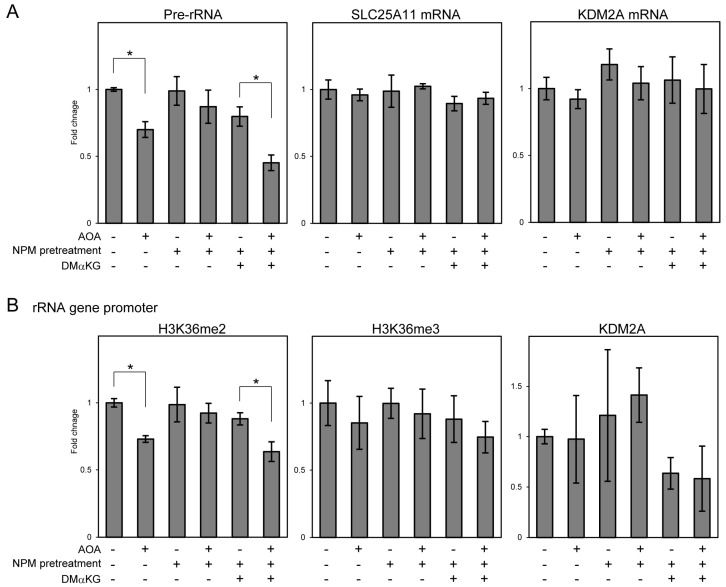
DMαKG restored the AOA-induced reduction in pre-rRNA and H3K36me2 levels in the rRNA gene promoter region inhibited by NPM pretreatment. (**A**) DMαKG addition restored the AOA-mediated decrease in pre-rRNA levels inhibited by NPM pretreatment. MCF-7 cells were treated with 1 mM AOA for 4 h. Additionally, 10 μM NPM pretreatment was performed 1 h before AOA treatment and/or 1 mM DMαKG was added 1 h before collection. The levels of pre-rRNA, SLC25A11 mRNA, and KDM2A mRNA were analyzed using RT-qPCR. Fold changes relative to non-treated cells are shown. (**B**) DMαKG restored the AOA-mediated decrease in H3K36me2 levels in the rRNA gene promoter region inhibited by NPM pretreatment. MCF-7 cells were treated under the same condition as that used in (**A**). The levels of H3K36me2, H3K36me2, and KDM2A in the rRNA gene promoter region were analyzed using the ChIP assay. Fold changes relative to non-treated cells are shown. Standard deviations are shown. *n* = 3., *; *p* < 0.05.

The above results suggest that NPM pretreatment inhibits KDM2A activity via SLC25A11 inhibition. Considering that SLC25A11 transports αKG into the cytoplasm, αKG may be involved in the inhibition of AOA-induced KDM2A activity by NPM pretreatment. Therefore, we examined the effect of treatment with DMαKG, a cell-permeable αKG, on KDM2A activity in NPM-pretreated/AOA-treated cells. Similarly to the results presented above, AOA treatment decreased pre-rRNA levels, but NPM pretreatment inhibited this reduction (Figure 7A). The addition of 1 mM DMαKG 1 h before sample collection restored the reduction in pre-rRNA levels by AOA treatment in NPM-pretreated cells (Figure 7A). The mRNA levels of SLC25A11 and KDM2A were not significantly altered under these conditions (Figure 7A). H3K36me2 levels in the rRNA gene promoter region also decreased upon AOA treatment, which was inhibited by NPM-pretreatment and restored by DMαKG addition (Figure 7B). H3K36me3 and KDM2A levels were not significantly changed under these conditions (Figure 7B). These results suggest that αKG is involved in the inhibition of AOA-induced KDM2A activity by NPM.

## 4. Discussion

The NADH shuttle transports the reducing equivalents of NADH to the mitochondria for ATP synthesis. In addition, the NADH shuttle regenerates NAD^+^, thereby maintaining glycolysis [3,4,28]. Therefore, MAS dysfunction likely causes a decrease in energy production and various responses, but the downstream reactions remain unclear. Specifically, the epigenetic control of these processes has not been well elucidated.

In previous studies, we demonstrated that rRNA transcription was reduced by KDM2A via H3K36me2 demethylation in the rRNA gene promoter region in response to treatment, such as with 2-DG and metformin, which decreased energy levels [14,15]. In these studies, AMPK activity was required for this reduction. During metformin treatment, intracellular succinate or αKG levels were also involved in modulating KDM2A activity [15]. These observations suggest that rRNA transcription control via demethylation by KDM2A responds to energy reduction but may be modulated by other factors. MAS contributes to energy production, and the process involves synthesis and transport of intermediates, including αKG. Here, we specifically focused on KDM2A-mediated control under MAS inhibition.

In this study, we showed that 4 h treatment with AOA, which is known to prevent MAS activity via AST/GOT inhibition, decreased cellular ATP levels and activated AMPK (Figure 1). AOA treatment decreased H3K36me2 levels in the rRNA gene promoter region and rRNA transcription via KDM2A (Figure 2). On the other hand, AOA inhibits aminotransferases, including AST/GOT, via pyridoxal 5′-phosphate inhibition [29]. In this study, induction of KDM2A activity under AOA treatment was also detected upon GOT1 knockdown (Figure S4). These results suggest that AOA-mediated inhibition of GOT1 contributes to the induction of KDM2A activity. These results further suggest that the regulation of rRNA genes via demethylation by KDM2A responds to energy reduction caused by MAS inhibition. We also considered the induction of KDM2A activity by AOA, similar to the response to 2-DG treatment observed in our previous study [14]. This supports the hypothesis that KDM2A activity is induced in the early stages of energy reduction caused by various factors.

On the other hand, NPM, which has been reported to inhibit SLC25A11 in MAS [23,24], also reduced ATP levels and activated AMPK (Figure 1); however, it did not induce the KDM2A activity observed in AOA-treated cells (Figure 3). Furthermore, AOA induced KDM2A-dependent proliferation inhibition, whereas NPM induced KDM2A-independent proliferation inhibition (Figure 4). These results suggest that induction of KDM2A activity, which suppresses rRNA transcription via demethylation, depends on the inhibition point. This suggests that responses may change depending on the cause of MAS dysfunction. Indeed, the clinical presentation of MAS deficiencies varies depending on the specific gene that is deficient [2,3].

AOA and NPM induced a decrease in ATP levels and activated AMPK (Figure 1). These responses were likely caused by a reduction in NADH shuttle activity. Defection of the NADH shuttle may induce NADH reduction stress, which is characterized by NADH accumulation [30]. NADH reduction stress is involved in various metabolic controls and subsequent metabolic reprogramming. For example, it affects the activity of NAD^+^-dependent enzymes such as SIRT family proteins. It was reported that SIRT6 directly binds to KDM2A and mono-ADP ribosylates it under DNA damaging conditions [31]. SIRT7 is involved in the activation of rRNA transcription via PAF53 deacetylation [32]. Such NAD^+^/NADH abnormalities may be involved in inducing KDM2A activity. However, differences in KDM2A induction capacity between AOA and NPM remain unclear.

Inhibition by AOA and NPM likely disrupts coupling reactions of MAS and causes transient differences in the amount and state of MAS intermediates. Under MAS reactions, cytoplasmic AST/GOT catalyzes the transfer of the amino group of aspartate to α-KG. Thus, α-KG is converted to glutamate, and aspartate is converted to oxaloacetate [3]. Therefore, inhibition of cytoplasmic AST/GOT by AOA may temporarily increase the levels of aspartate and α-KG in the cytoplasm. Mitochondrial AST/GOT catalyzes the reverse reaction of cytoplasmic AST/GOT. This reaction transfers the amino group of glutamate to oxaloacetate [3]. Therefore, AOA may temporarily increase the concentration of glutamate and oxaloacetate in the mitochondria. On the other hand, NPM inhibits the mitochondrial malate/αKG exchange carrier SLC25A11. SLC25A11 transports αKG from mitochondria to the cytoplasm and malate from the cytoplasm to the mitochondria [3]. Therefore, NPM may reduce αKG levels in the cytoplasm and malate levels in the mitochondria. These differences between AOA and NPM may affect the inductive activity of KDM2A under MAS dysfunction. Indeed, changes in individual components of MAS appear to have different effects on the intracellular amounts of intermediates and other metabolic pathway products [1,4]. However, transient changes are not well understood because detection methods have not been established. Real-time detection methods may help close this gap.

NPM pretreatment inhibited the reduction in H3K36me2 levels in the rRNA gene promoter region and rRNA transcription induced by AOA (Figure 5). SLC25A11 knockdown also inhibited this reduction (Figure 6). These results suggest that SLC25A11 is involved in KDM2A activity induced by AOA. The demethylation activity of jmjC-type enzymes, including KDM2A, requires a side reaction from αKG to succinate [9]. The transport of αKG by SLC25A11 may be involved in the KDM2A activity under MAS inhibition by AOA. DMαKG, a cell-permeable αKG, has been reported to be cleaved into αKG by cytoplasmic esterase [33]. DMαKG treatment restored KDM2A activity inhibited by NPM pretreatment in AOA-treated cells (Figure 7). These results suggest that the effect of NPM on cytoplasmic α-KG levels may be involved in the inhibition of KDM2A activity for rRNA transcription. SLC25A21, an αKG transporter in mitochondria, has been reported to affect the activity of the jmjC-type DNA demethylase TET in KRAS-mutant colorectal cancer [34]. Although the mechanism by which αKG is supplied to jmjC-type enzymes is not well understood, these findings suggest the existence of a supply pathway from the mitochondria. Elucidating this pathway is expected to contribute to our mechanistic understanding of how metabolic abnormalities are reflected in genetic regulation.

A limitation of this study is that we were unable to accurately evaluate changes in MAS intermediates in the cytoplasm and mitochondria caused by MAS inhibition. Future studies that clarify changes in αKG transport owing to SLC25A11 inhibition and elucidate the effect of transported αKG on KDM2A activity will reveal new aspects of MAS function.

## 5. Conclusions

This study revealed that epigenetic regulation of rRNA transcription via KDM2A is induced by MAS inhibition. Analysis using two MAS inhibitors, AOA and NPM, suggested that the induction capacity of KDM2A activity differs depending on the inhibition point. This difference was suggested to involve SLC25A11, the αKG transporter in MAS.

## Data Availability

All data generated or analyzed in this study are included in this published article and its Supplementary Materials files.

## Supplementary Materials

The following supporting information can be downloaded at: https://www.mdpi.com/article/10.3390/cells14211655/s1, Figure S1: ATP levels after AOA and NPM treatment; Figure S2: Levels of H3K36me2, H3K36me2, and KDM2A in the rRNA gene body region in KDM2A knockdown cells treated with 1 mM AOA for 4 h; Figure S3: AMPK inhibitor prevents the reduction of rRNA transcription by AOA; Figure S4: GOT1 knockdown reduced H3K36me2 levels in the rRNA gene promoter region and rRNA transcription via KDM2A; Figure S5: Levels of H3K36me2, H3K36me2, and KDM2A in the rRNA gene body region in cells treated with NPM.
