# Supplementary material for: SLC25A11 Is Associated with KDM2A-Dependent Reduction in rRNA Transcription Induced by Aminooxyacetic Acid
Source: Cells. 2025 Oct 22;14(21):1655. doi: 10.3390/cells14211655 (PMC12609500; doi:10.3390/cells14211655)
Supplement: Supplementary file 1 [file cells-14-01655-s001.zip › cells-3883959-supplementary.pdf]

Supplementary Materials

SLC25A11 is associated with KDM2A-dependent reduction in rRNA transcription induced by aminooxyacetic acid

Yuji Tanaka*, Nagisa Miyazawa, Yuuki Toba
Laboratory of Transcriptional Regulation, Faculty of Pharmacy, Takasaki University of Health and Welfare, 60 Nakaorui-machi, Takasaki-shi, Gunma, 370-0033, Japan.
*Correspondence: ytanaka@takasaki-u.ac.jp

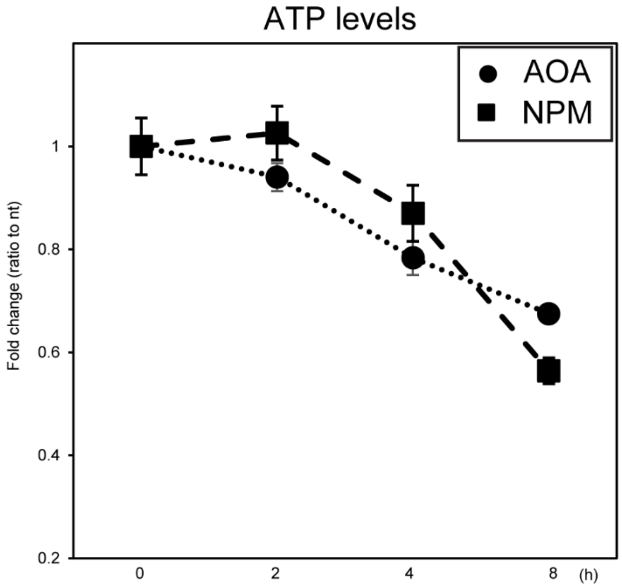

Figure S1. ATP levels after AOA and NPM treatment

ATP levels of MCF-7 cells treated with 1 mM AOA or 10 μM NPM at the indicated time were measured. Relative ATP levels at each time point are shown as fold changes relative to the ATP levels at 0 h. Standard deviations are shown. *n*=4.

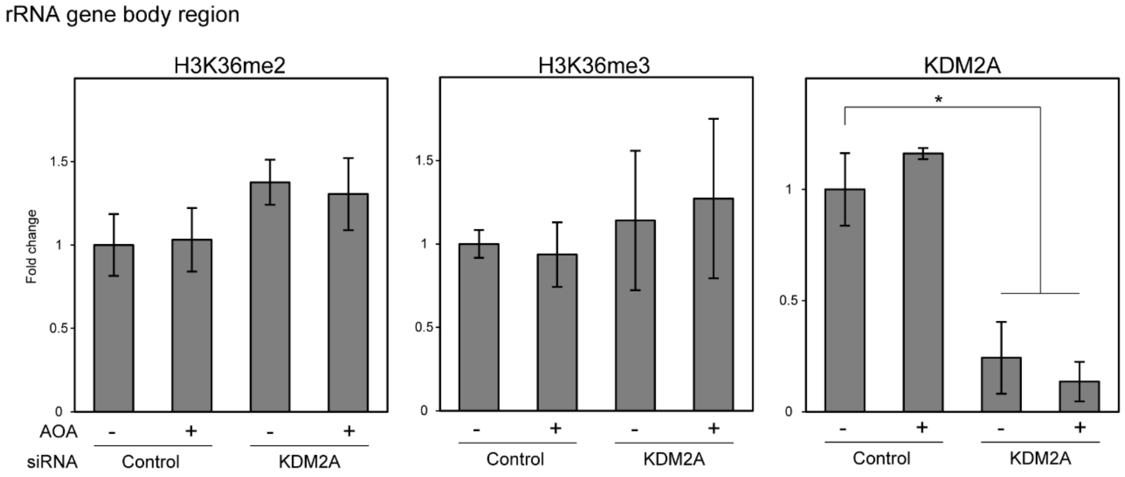

Figure S2. Levels of H3K36me2, H3K36me2, and KDM2A in the rRNA gene body region in KDM2A knockdown cells treated with 1 mM AOA for 4 h

The ChIP products shown in Fig. 2B were analyzed using specific primers to detect rRNA gene body regions. Briefly, MCF-7 cells were transfected with control siRNA (control) or siRNA against KDM2A (KDM2A). Cells were treated with or without 1 mM AOA. Levels of H3K36me2, H3K36me2, and KDM2A in the rRNA gene body region were analyzed using the ChIP assay. Fold changes relative to the control siRNA without AOA treatment are shown. Standard deviations are shown. n=3, *; p<0.05.

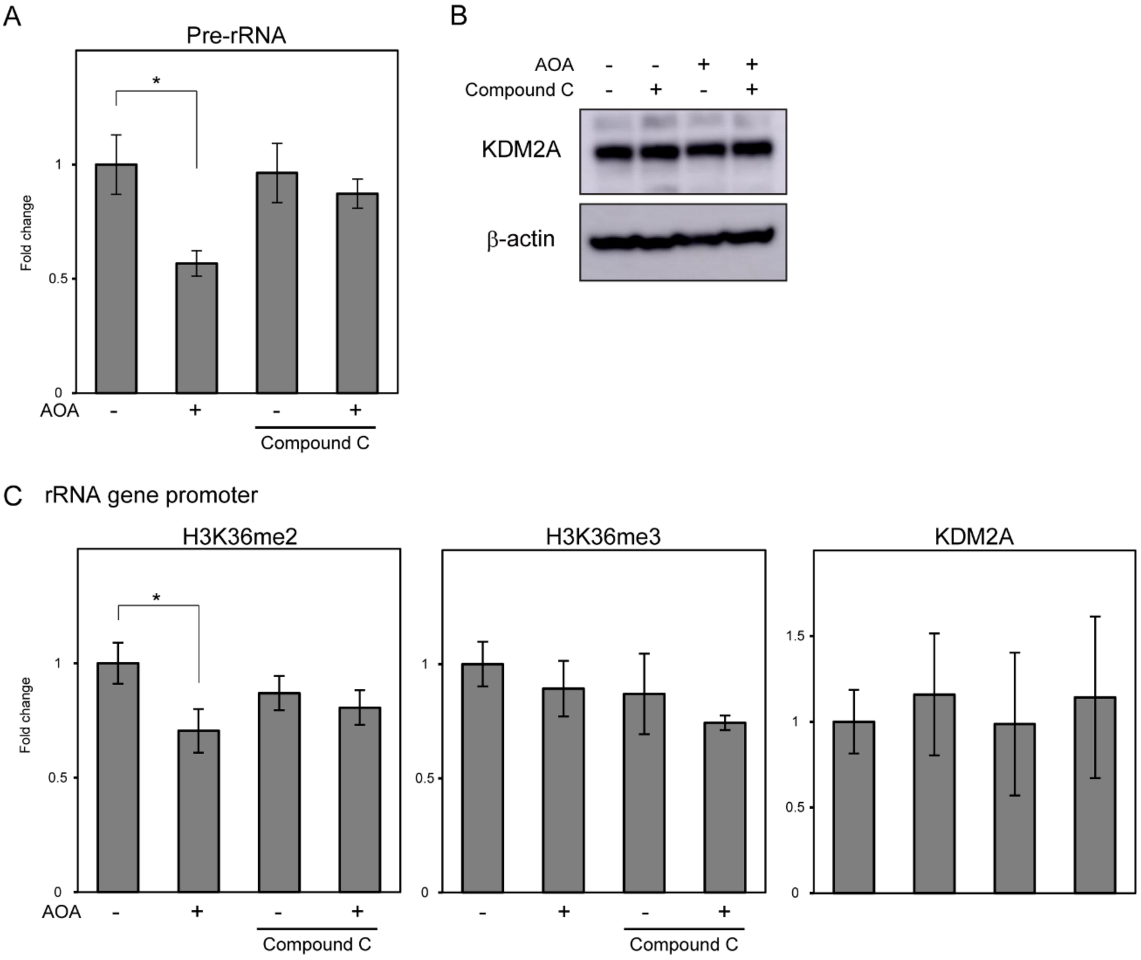

Figure S3. AMPK inhibitor prevents the reduction of rRNA transcription by AOA

(A) Compound C inhibited AOA-induced reduction in pre-rRNA levels. MCF-7 cells were treated with 1 mM AOA and with or without 10 µM Compound C, an inhibitor for AMPK, for 4 h. Pre-rRNA levels were analyzed using RT-qPCR. Fold changes relative to non-treated cells are shown. (B) Protein levels of KDM2A in cells treated with AOA or Compound C. Whole cell lysates of MCF-7 cells treated under the same conditions as those in (A) were analyzed by immunoblotting with specific antibodies against KDM2A and β-actin. (C) Compound C inhibited the AOA-induced decrease in H3K36me2 levels in the rRNA gene promoter region. MCF-7 cells treated under the same conditions as those in (A) were analyzed. H3K36me2, H3K36me3, and KDM2A levels in the rRNA gene promoter region were analyzed using the ChIP assay. Fold changes relative to non-treated cells are shown. Standard deviations are shown. n=3, *; p<0.05.

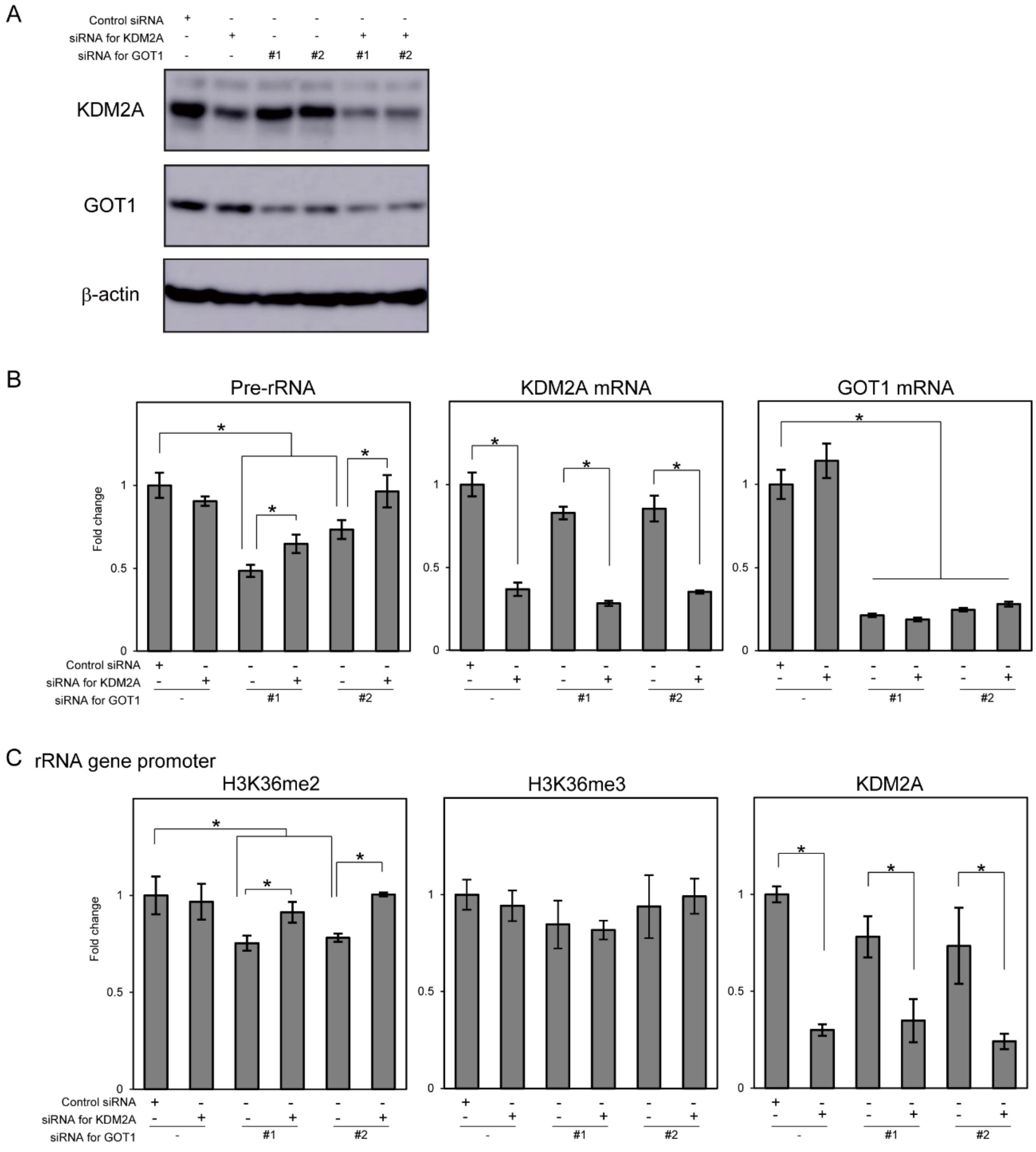

Figure S4. GOT1 knockdown reduced H3K36me2 levels in the rRNA gene promoter region and rRNA transcription via KDM2A

(A) Levels of GOT1 and KDM2A proteins in knockdown cells. MCF-7 cells were transfected with control siRNA, siRNA for KDM2A, and siRNA for GOT1 #1 and #2 under the indicated conditions. Whole cell lysates were collected from these cells and analyzed using immunoblotting with specific antibodies against KDM2A, GOT1, and β-actin. (B) GOT1 knockdown reduces pre-rRNA levels in dependence of KDM2A. Pre-rRNA, KDM2A mRNA, and GOT1 mRNA levels in MCF-7 cells transfected with control siRNA or siRNA for GOT1 #1, #2, or KDM2A under the indicated conditions were analyzed using RT-

qPCR. Levels of pre-rRNA and KDM2A mRNA were normalized with β-actin mRNA levels and are shown as fold changes relative to control cells. (**C**) GOT1 knockdown reduces H3K36me2 levels in the rRNA gene promoter region in dependence of KDM2A. H3K36me2, H3K36me3, and KDM2A levels in the rRNA gene promoter region under same condition as those in (**B**) were analyzed using the ChIP assay. Levels of H3K36me2 and H3K36me3 were normalized to total histone H3 levels and are shown as fold changes relative to control cells. Standard deviations are shown. n=3., *; p<0.05.

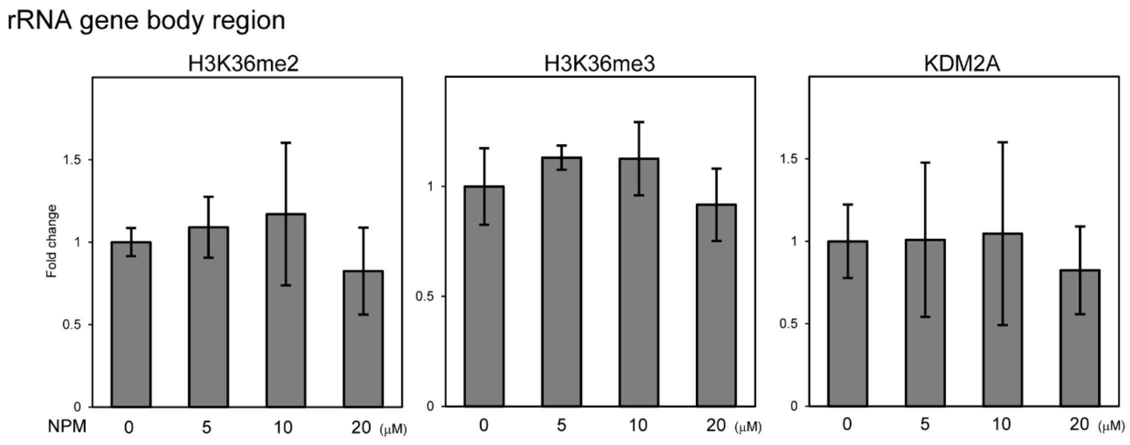

Figure S5. Levels of H3K36me2, H3K36me2, and KDM2A in the rRNA gene body region in cells treated with NPM

The ChIP products shown in Fig. 3B were analyzed using specific primers to detect rRNA gene body regions. Briefly, MCF-7 cells were treated with NPM at the indicated concentrations for 4 h. H3K36me2, H3K36me2, and KDM2A levels in the rRNA gene body were analyzed using the ChIP assay. Fold changes relative to non-treated cells are shown. Standard deviations are shown. n=3.

Supplementary materials and methods

Chemicals

Compound C (IN Solution™ AMPK inhibitor, #171261) was purchased from Merck, Darmstadt, Germany.

Antibodies

Anti-GOT1 (#14886-1-AP) was purchased from Proteintech Group, Inc., Rosemont, IL, USA.

Primers

The primers for amplification of the human rRNA gene body region (+12885 to +12970 from the transcription start site) used for the ChIP assay were 5'-ACCTGGCGCTAAACCATTCGT-3' and 5'-GGACAAACCCTTGTGTCGAGG-3'. The primers were the same as those used in a previous study [1]. Primers for human GOT1 mRNA were 5'- ATGGCACCTCCGTCAGTCT-3' and 5'-AGTCATCCGTGCGATATGCTC -3'. The primer sequences were obtained from PrimerBank [2].

References

1. Tanaka, Y.; Okamoto, K.; Teye, K.; Umata, T.; Yamagiwa, N.; Suto, Y.; Zhang, Y.; Tsuneoka, M. JmjC enzyme KDM2A is a regulator of rRNA transcription in response to starvation. *EMBO J* 2010, *29*, 1510-1522. doi:10.1038/emboj.2010.56.
2. Spandidos, A.; Wang, X.; Wang, H.; Seed, B. PrimerBank: a resource of human and mouse PCR primer pairs for gene expression detection and quantification. *Nucleic Acids Res* 2010, *38*, D792-799. doi:10.1093/nar/gkp1005.
